# Ovarian Abnormalities in the Staggerer Mutant Mouse

**DOI:** 10.1100/tsw.2005.87

**Published:** 2005-08-24

**Authors:** Jean-Marie Guastavino, Salima Boufares, Wim E. Crusio

**Affiliations:** ^1^ Laboratoire d'Ethologie et de Sociobiologie, Université de Paris XIII, Paris, France; ^2^ Service des Relations Internationales, Université Henri Poincaré-Nancy I, 24-30 rue Lionnois, BP 60120, 54003 Nancy Cedex, France; ^3^ Laboratoire de Neurosciences Cognitives, CNRS UMR 5106, Avenue des Facultés, 33405 Talence, France

**Keywords:** staggerer, ovary, cerebellum, reproduction, fertility, neurological mutant

## Abstract

Disturbances in several reproductive functions of the staggerer cerebellar mutant mouse have been observed. In this study, reproductive efficiency of *staggerer* mice was compared to normal mice by recording the number of pups produced and the number of oocytes occurring. It was found that *staggerer* mothers produced smaller litters than controls and the number of oocytes produced in their ovaries was reduced by the *staggerer* mutation. These results indicate a pleiotropic effect on fertility of the Rora^sg^ gene underlying the cerebellar abnormalities of the *staggerer* mutant.

## INTRODUCTION

The target of the *staggerer* mutation was first described as a cerebellar atrophy resulting in alteration of gait and body balance[1,2]. The staggerer mutation (original gene symbol *sg*, now $Rora^{sg}$; chromosome 9) is a deletion in the gene coding for the retinoic acid–related orphan receptor alpha (RORα), a member of the nuclear hormone–receptor superfamily[3]. In normal mice, this receptor interacts with the thyroid pathway to regulate the development of Purkinje cells[3]. Indeed, the observed motor deficits seem to be primarily due to a defect in the early postnatal development of the Purkinje cells[4]. Other research has indicated several other anatomical and functional abnormalities including alterations of the immune system[5,6,7], reactions to novelty[8,9,10,11], spatial learning[12,13,14], cold adaptation[15,16], olfactory bulb characteristics[17,18], and reproductive behavior[19,20]. Reproduction is often altered in mutants and this appears also to be the case for the *staggerer* mutation, where both male and female reproductive capacity are highly abnormal[20,21,22]: Few *staggerer* males were sexually active, often showing a constant penile erection[22]. Over a 15-year period of breeding in our laboratory, we have found that about 2 out of 3 *staggerer* females reproduce. Nevertheless, although able to reproduce, *staggerer* females showed delayed onset of puberty, abnormalities in the estrous cycle[23], and a marked acceleration of the reproductive aging process[20]. The abnormal temporal pattern of the female reproductive functions prompted us to investigate systematically the fertility of the *staggerer* female by the number of pups produced and by the number of oocytes present in the ovaries.

## METHODS

The animals used in this study were mutant and nonmutant female mice on a congenic C57BL/6J background, bred in our laboratory since 1980. Mutants were $Rora^{sg}/Rora^{sg}$ and were the offspring of intercrosses between $+/Rora^{sg}$ heterozygotes. Since the mutation is recessive, and homozygous and heterozygous animals cannot be distinguished from one another phenotypically, the control group of nonmutants consisted of the +/+ or $+/Rora^{sg}$ offspring of the same matings. The animals were housed in standard mouse cages with a 12:12 light-dark cycle with lights on at 9 a.m., 24°C, and with free access to solid and soft food and water. The females were weaned at 35 days of age and thereafter maintained in groups of 6 females.

To compare the number of pups produced, we used 15 *staggerer* and 15 control females. A first crossing with normal males was performed when the animals were 2–4 months old. Later, when the animals were 4–6 months old, a second crossing was performed. For both crossings, the number of pups born was counted.

For the study of the ovaries, 8 primiparous *staggerer* and 8 control females were used. When the animals were 3.5–5 months old, and about 2–4 weeks after parturition, the ovaries were removed. Only one ovary per female was used. The ovarian tissues were fixed for 24 h in a solution of Bouin Holland and washed with water for at least 1 day to eliminate excess picric acid. Specimens were then dehydrated in a graded series of ethanol and finally washed in butanol. After embedding in paraffin, transverse 5-µm-thick sections were cut and mounted on slides.

The slices were colored by a Schiff periodic acid solution (1%) for 10 min, rinsed repeatedly before being treated with Schiff reagent, and thereafter rinsed again. The slices were thereafter treated with hematoxyline of Groat for 10 min. After prolonged rinsing (15 min), the slices were subjected to a final dehydration procedure and mounted on glasses using a synthetic resin (Eukitt, Kindler, Freiburg, Germany) before being checked under the microscope (Reichert, Polyvar).

## RESULTS AND CONCLUSIONS

As shown in Table l, the *staggerer* mothers produced significantly fewer pups per litter than the controls both after the first and the second mating. Also, the numbers of oocytes counted was significantly lower in the *staggerer* females than in the controls ($p < 0.05$). Comparing the number of pups between the first and second mating, a statistically significant increase was seen both in the *staggerer* females ($p < 0.01$) and in the controls ($p < 0.01$).

The present results show that reproductive function is one of the targets of the *staggerer* mutation as evidenced both by the number of pups produced and the number of oocytes per ovary. These observations are interesting in the light of several other indications of reproductive deficits in the *staggerer* mouse as mentioned in the Introduction. At this point, it is difficult to explain these deficits, although it is unlikely that they are a direct consequence of the cerebellar dysfunction in these animals. One possibility is that the reduced fertility could be due to a pleiotropic effect of the $Rora^{sg}$ allele. Alternatively, this deficit could be due to secondary effects of other disorders produced by this gene. We previously reported the involvement of the cerebellum in the organization of food intake and digestive functions[16] and it is a well-established fact that undernutrition may be followed by alterations in reproductive functions. Further studies are needed to define the precise mechanisms involved in mediating the alterations.

**TABLE 1**
**The Number of Pups (mean ± SEM) Produced After the First and Second Matings of 15 *Staggerer* and 15 Normal Mice and Number of Oocytes Produced After the First Mating of 8 *Staggerer* and 8 Controls**

| | ***Staggerer*** | **Control** | ***p**** |
|---|---|---|---|
| | Number of pups per litter | | |
| Mating 1 | 6.76 ± 0.41 | 8.53 ± 0.65 | <0.02 |
| Mating 2 | 8.31 ± 0.37 | 10.40 ±0.68 | <0.01 |
| | Number of oocytes per ovary | | |
| | 5.25 ± 0.37 | 6.75 ±0.37 | <0.05 |

*Mann-Whitney U-Test[24]

## ACKNOWLEDGMENTS

We thank the Centre National de la Recherche Scientifique (CNRS, Paris, France), the Swedish Natural Science Research Council (NFR, Stockholm, Sweden), and the Swedish-French Association for Research (ASFR, Stockholm, Sweden) for support.
